# Matrix Metalloproteinase 7 Mediates Epithelial–Mesenchymal Transition to Promote Liver Fibrosis Through E-cadherin/β-catenin Pathway in Biliary Atresia

**DOI:** 10.3390/ijms27052209

**Published:** 2026-02-26

**Authors:** Liying Rong, Jingfeng Tang, Xiangyang Li, Mengxin Zhang, Shuiqing Chi, Yun Zhou, Xi Zhang, Guoqing Cao, Yibo Li, Shaotao Tang

**Affiliations:** 1 Department of Pediatric Surgery, Union Hospital, Tongji Medical College, Huazhong University of Science and Technology, Wuhan 430022, China; rongliy@foxmail.com (L.R.);; 2 Department of Traumatology and Emergency Surgery, Union Hospital, Tongji Medical College, Huazhong University of Science and Technology, Wuhan 430022, China; tangjingfeng86@126.com

**Keywords:** biliary atresia, matrix metalloproteinase-7, epithelial–mesenchymal transformation, liver fibrosis, chronic biliary atresia model

## Abstract

Biliary atresia (BA) is characterized by rapidly progressive hepatic fibrosis with unclear mechanisms. This study aimed to investigate the role of matrix metalloproteinase 7 (MMP7) in this process and its potential for targeted therapy. Serum and liver tissue samples from BA patients were collected to analyze the correlation between MMP7 and liver fibrosis. Gene set enrichment analysis (GSEA) based on GEO datasets was performed to explore MMP7-associated biological processes. Clinical samples were further used to examine the relationship between MMP7 and epithelial–mesenchymal transition (EMT) in biliary epithelial cells (BECs). The effects of MMP7 on BECs and the underlying mechanisms were validated in vitro. Finally, the profibrotic effects and therapeutic potential of MMP7 were explored in chronic BA mice. Results showed that MMP7 was positively correlated with liver fibrosis in BA patients. GSEA revealed that MMP7 was most significantly associated with EMT, which was further validated by EMT scoring in intrahepatic BECs of patients. In vitro, MMP7 induced EMT in BECs by cleaving E-cadherin and promoting β-catenin nuclear translocation. Blockade of MMP7 alleviated EMT and liver fibrosis in BA mice. In conclusion, MMP7 promotes liver fibrosis in BA by driving EMT via the E-cadherin/β-catenin pathway, and targeting MMP7 demonstrates anti-fibrotic effects.

## 1. Introduction

Biliary atresia (BA), a devastating disease of infancy, is characterized by obstructive jaundice and progressive fibro-inflammatory cholangiopathy. Unlike other hepatic or biliary diseases, liver fibrosis progresses more rapidly in BA. Untreated patients typically do not survive beyond the age of 2 due to cholestasis and cirrhosis [1,2]. The Kasai portoenterostomy (KPE) is currently the primary treatment for patients with BA, which involves resection of the extrahepatic obstructed bile ducts and reconstruction of bile drainage via a Roux-en-Y anastomosis. However, the outcomes of the procedure are variable [3]. In some patients, despite the clearance of jaundice in the early postoperative period, intrahepatic fibrotic progression continues [4]. Current understanding of liver fibrosis in BA remains limited, with significant gaps in the knowledge of its progression mechanisms. Existing postoperative adjuvant therapies, such as corticosteroids and ursodeoxycholic acid, show limited efficacy in delaying the advancement of liver fibrosis, necessitating liver transplantation to ensure long-term survival [3]. Therefore, there is an urgent need to further investigate the mechanisms underlying liver fibrosis progression in BA and to identify novel therapeutic targets.

As a member of the zinc-dependent endopeptidase family, matrix metalloproteinase 7 (MMP7) is essential for the degradation of various components of the extracellular matrix (ECM). Additionally, it cleaves a range of non-ECM substrates, including E-cadherin, tumor necrosis factor-α (TNF-α), and Fas ligand, thereby participating in various pathophysiological processes [5,6]. MMP7 has drawn considerable interest in BA following its identification as a highly specific diagnostic serum biomarker through SOMAscan screening [7,8,9]. Study has shown that the administration of an MMP7 antibody or inhibitor (Batimastat) prevented bile duct obstruction and alleviated liver inflammation and injury in BA mouse models, suggesting a potential role of MMP7 in BA pathogenesis [7]. In pulmonary and renal fibrosis, MMP7 has been demonstrated to exert pro-fibrotic effects [10,11]. Although recent studies have indicated a correlation between MMP7 levels and the degree of liver fibrosis at the time of KPE in BA patients, the pro-fibrotic mechanisms of MMP7 in BA remain unexplored [12,13,14,15]. Notably, our previous serological follow-up study revealed that MMP7 levels remain persistently elevated in some BA patients even after KPE, and these patients tend to have a poorer prognosis [16]. This observation suggests a continuing pro-fibrotic role of MMP7 after surgery and highlights its potential as a therapeutic target.

In this study, we further validated the association between intrahepatic MMP7 and liver fibrosis in specimens from BA patients. We then performed Gene Set Enrichment Analysis (GSEA) and confirmed the epithelial–mesenchymal transition (EMT) pathway as a potential downstream pathway of MMP7 and elucidated the specific mechanisms using clinical samples and human intrahepatic biliary epithelial cells (HIBEpiCs) in vitro. Finally, we utilized a BA mouse model to explore the therapeutic potential of targeting MMP7 against liver fibrosis.

## 2. Results

### 2.1. Levels of MMP7 Were Related to Liver Fibrosis in BA Patients

**Figure 1:**
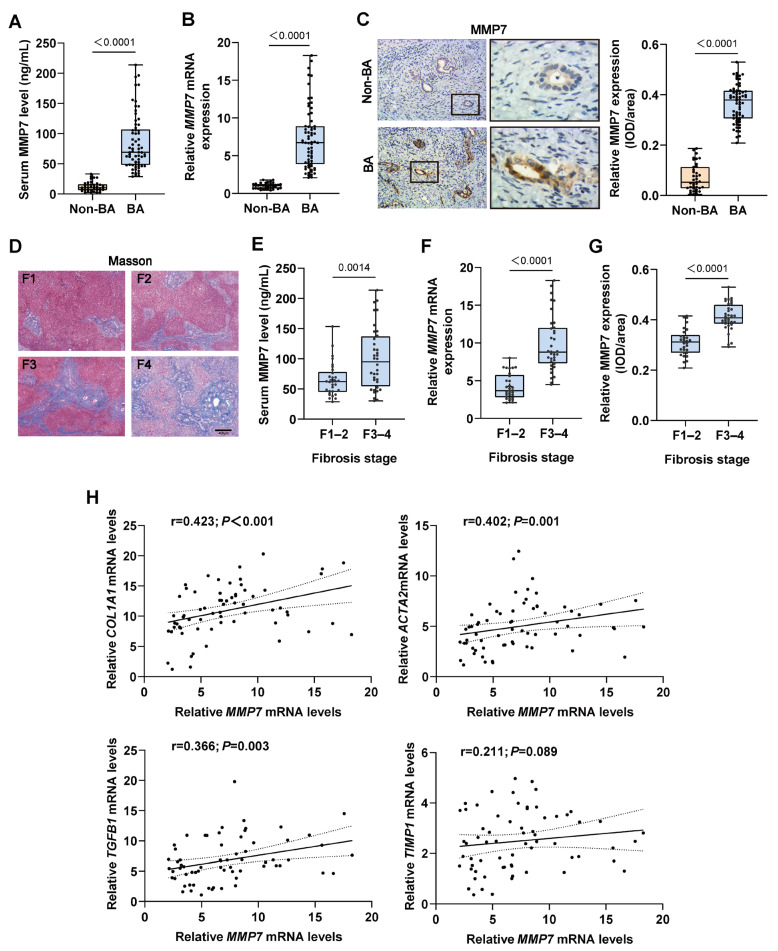
Increased MMP7 in BA patients correlated with the progression of liver fibrosis. Serum and liver samples of patients with non-BA (*n* = 39) disease and BA (*n* = 66) were collected. (**A**) Serum MMP7 levels were determined by ELISA. (**B**) Relative *MMP7* mRNA levels in livers of BA and non-BA patients. (**C**) Representative images of immunostaining for MMP7 and quantitative analysis of MMP7 staining in liver tissue. Magnification: left panel: ×100; right panel: ×400. (**D**) Representative Masson trichrome staining images of METAVIR fibrosis stages (F1–F4) in BA patients. Scale bar: 400 μm. (**E**–**G**) Serum MMP7 levels, relative *MMP7* mRNA levels and the expression intensity of MMP7 in liver of BA patients with moderate (F0–F2) or advanced liver fibrosis (F3–F4). (**H**) Spearman’s rank correlation analysis of *MMP7* expression level with fibrotic marker genes, including *COL1A1*, *ACTA2*, *TGFB1*, and *TIMP1*.

Compared to non-BA patients, both serum levels and hepatic mRNA expression of MMP7 were elevated in BA patients (Figure 1A,B). The immunostaining showed that MMP7 exhibited elevated expression levels in the BECs of BA patients, with the intensity of MMP7 expression being significantly greater (Figure 1C). To further determine whether MMP7 expression was associated with BA-related liver fibrosis, we classified the degree of liver fibrosis in 66 BA patients into four grades according to the METAVIR score (Figure 1D; Table 1). Serum concentrations of MMP7 were higher in patients of grades F3 and F4 compared with those of grades F1 and F2 (Figure 1E). Consistently, both hepatic mRNA and protein expression of MMP7 was higher in the severe fibrosis group (Figure 1F,G). The mRNA expression of hepatic fibrosis marker genes was significantly elevated in patients with BA, which including collagen type I alpha 1 (*COL1A1*), alpha actin 2 (*ACTA2*), transforming growth factor beta 1 (*TGFB1*), and tissue inhibitor of metalloproteinases-1 (*TIMP1*) (Supplementary Figure S1). Correlation analyses revealed a positive association between hepatic MMP7 mRNA expression and the expression levels of *COL1A1* (r = 0.423; *p* < 0.001), *ACTA2* (r = 0.402; *p* = 0.001), and *TGFB1* (r = 0.366; *p* = 0.003). No significant correlation was observed with *TIMP1* (r = 0.211; *p* = 0.089; Figure 1H). Additionally, in BA patients, higher inflammatory grades (A2 and A3) were associated with significantly elevated MMP7 levels in both serum and liver tissue (Supplementary Figure S2).

### 2.2. MMP7-Associated Pathways in BA

**Figure 2:**
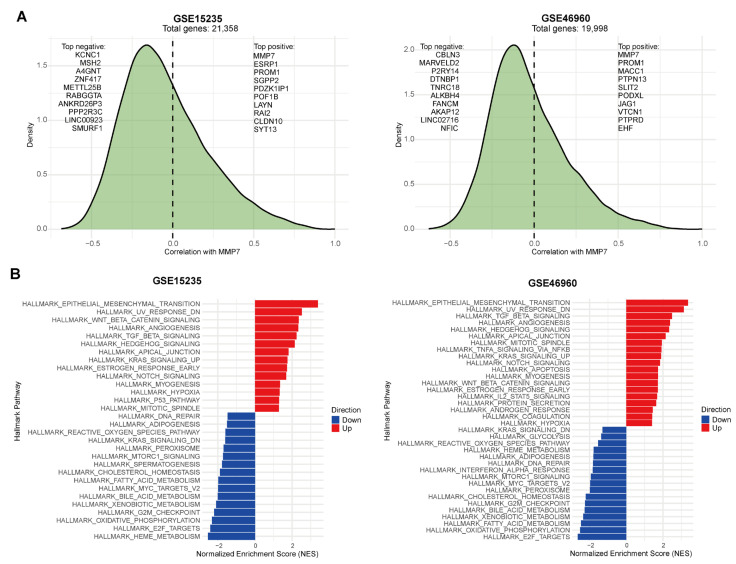
MMP7 gene correlation analysis and pathway enrichment in BA. (**A**) Distribution of correlation coefficients between MMP7 expression and all other genes across two independent BA datasets (GSE15235 and GSE46960). (**B**) GSEA results using gene lists ranked by correlation with MMP7.

To elucidate the biological processes linked to MMP7 in BA, we performed a correlation analysis of MMP7 expression with all other genes across two independent BA datasets (GSE15235 and GSE46960) from the Gene Expression Omnibus (GEO) database [https://www.ncbi.nlm.nih.gov/geo/ (accessed on 11 March 2023)]. The resulting distribution of correlation coefficients is shown in Figure 2A. The ranked gene lists based on their correlation with MMP7 were subsequently used for Gene Set Enrichment Analysis (GSEA) based on the HALLMARK gene set in the MSigDB database. Notably, the EPITHELIAL_MESENCHYMAL_TRANSITION pathway emerged as the most positively enriched signature associated with high MMP7 expression in both cohorts (Figure 2B). Furthermore, several other pathways, including WNT_BETA_CATENIN_SIGNALING, TGF_BETA_SIGNALING, and ANGIOGENESIS, were also significantly enriched (Figure 2B, Supplementary Tables S1 and S2). These consistent results implicate MMP7 within a network of key processes central to tissue remodeling and fibrogenesis in BA.

### 2.3. EMT Occurred in BECs of BA Patients and Correlates with MMP7 Expression and Liver Fibrosis

**Figure 3:**
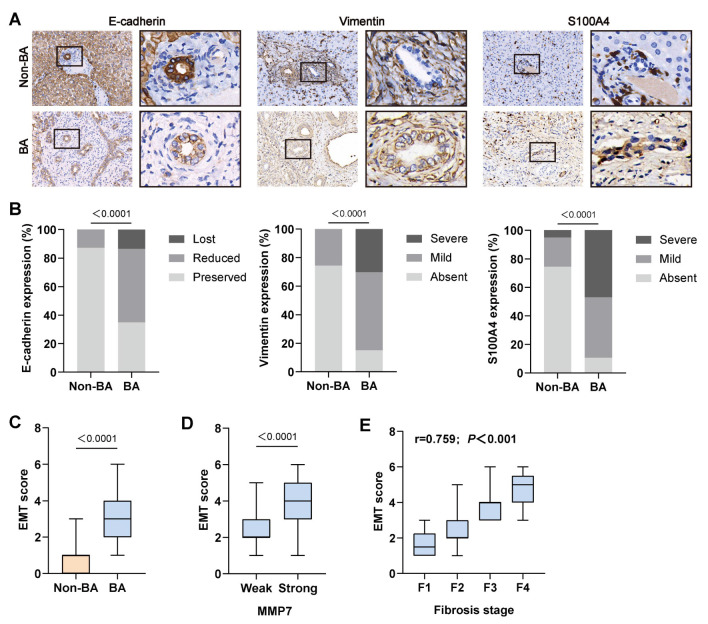
EMT scores were associated with MMP7, liver fibrosis and survival in BA patients. Liver samples of patients with non-BA (*n* = 39) disease and BA (*n* = 66) were collected. (**A**) Representative image of immunostaining for EMT-related protein expression (including E-cadherin, Vimentin, S100A4) in liver biopsies samples. Magnification: **left panel**: ×100; **right panel**: ×400. (**B**) Percentage of samples according to the intensity and extension of EMT-related protein immunostaining in cholangiocytes from two groups. (**C**) EMT scores of cholangiocytes in non-BA and BA patients. (**D**) EMT scores in two subgroups divided by MMP7 expression intensity in BA patients. (**E**) Correlation analysis of fibrosis stage EMT scores in BA patients. Spearman’s rank correlation.

BA is a disorder affecting both intra- and extrahepatic biliary trees. Therefore, based on the results of GSEA, we further explored the epithelial–mesenchymal transition (EMT) in intrahepatic biliary epithelial cells (BECs). Based on methods described in a previous study [17], we examined the expression of EMT-related markers by semiquantitative immunohistochemical staining. The epithelial marker E-cadherin was downregulated in BECs, whereas the mesenchymal markers vimentin and S100A4 were upregulated (Figure 3A,B). By summing the staining scores of these three markers, we obtained an EMT score to indicate the overall EMT level in BECs. EMT scores were higher in BA group compared to non-BA group (Figure 3C). Based on the intensity of MMP7 staining in BECs, BA patients were categorized into two groups: strong and weak. The EMT scores were higher in the strong group than in the weak group (Figure 3D). Furthermore, correlation analysis demonstrated a positive correlation between the EMT score and the liver fibrosis stage in BA group (r = 0.759; *p* < 0.001; Figure 3E).

### 2.4. MMP7 Induced EMT in BECs by Activating the E-cadherin/β-catenin Pathway

**Figure 4:**
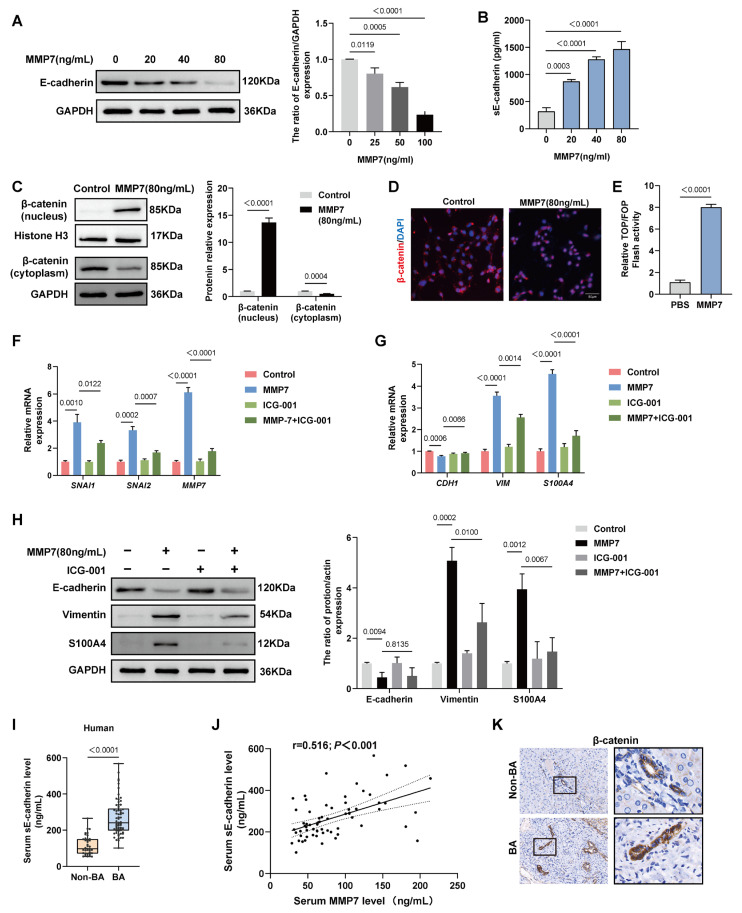
MMP7 promoted EMT in an E-cadherin/β-catenin pathway-dependent manner. (**A**) Protein expression of E-cadherin in HIBEpiCs after treatment with different concentrations of MMP7 for 24 h. (**B**) Level of sE-cadherin in the cell culture supernatant of HIBEpiCs after MMP7 treatment. (**C**) Western blot analysis of β-catenin protein in the nucleus or cytoplasm of HIBEpiCs. (**D**) Immunofluorescence staining shows the subcellular localization of β-catenin in HIBEpiCs. Scale bar:50 μm. (**E**) TOP/FOP-Flash luciferase activity of MMP7-treated cells. (**F**) Effect of the MMP7 and ICG-001 on β-catenin downstream target genes expression (including *SNAI1*, *SNAI2*, *MMP7*) in HIBEpiCs. (**G**,**H**) Effect of the MMP7 and ICG-001 on mRNA and protein expression of EMT-related markers (including E-cadherin, Vimentin, S100A4) in HIBEpiCs. (**I**) ELISA analysis of serum level of sE-cadherin in patients. (**J**) Spearman’s rank correlation analysis of serum sE-cadherin and serum MMP7 in BA patients. (**K**) Representative images of immunostaining of β-catenin in cholangiocytes of non-BA and BA patients. Magnification: **left panel**: ×100; **right panel**: ×400. (**C**–**H**) MMP7 concentration: 80 ng/mL; ICG-001 concentration: 25 μM. Data are presented as mean ± SD.

To investigate the mechanism by which MMP7-induced EMT in BECs, we analyzed the effects of recombinant human MMP7 protein on HIBEpiCs. Different concentrations of MMP7 (0, 20, 40, and 80 ng/mL) were added to the HIBEpiCs culture for 24 h. The Western blot analysis showed a concentration-dependent decrease in the levels of full-length E-cadherin protein within the cells (Figure 4A). In supernatants, the concentrations of soluble E-cadherin (sE-cadherin) increased with increasing MMP7 concentration as measured by ELISA (Figure 4B). These findings suggested that MMP7 induced E-cadherin ectodomain shedding in HIBEpiCs. Since the cytoplasmic domain of E-cadherin associates with β-catenin, disruption of the E-cadherin/β-catenin complex can facilitate the β-catenin nuclear translocation and enhance its transcriptional activity [18]. We further conducted an analysis of β-catenin localization in different cellular compartments utilizing Western blot and immunofluorescence. The stimulation of MMP7 (80 ng/mL, same as below) resulted in the nuclear accumulation of β-catenin, accompanied by a corresponding decrease in the levels of cytoplasmic β-catenin (Figure 4C,D). Accordingly, the transcriptional activity of β-catenin was assessed using Top/Fop-Flash luciferase activity assays, which indicated that treatment with MMP7 enhanced the transcriptional activity of β-catenin in HIBEpiCs (Figure 4E). Next, we evaluated the impact of MMP7 on the expression of target genes downstream of β-catenin. The treatment of MMP7 enhanced the mRNA expression of β-catenin target genes, including the EMT-related transcription factors SNAI1 and SNAI2, as well as MMP7 itself (Figure 4F). Co-treatment with the β-catenin inhibitor ICG-001 (25 μM) attenuated MMP7-mediated mRNA induction of *SNAI1*, *SNAI2*, and *MMP7* (Figure 4F). These results prompted us to examine whether MMP7 induces EMT by activating β-catenin. Treating HIBEpiCs with MMP7 for 24 h resulted in EMT, as demonstrated by a reduction in both mRNA and protein levels of E-cadherin, and an elevation in the levels of vimentin and S100A4 (Figure 4G,H). The increased mRNA and protein expression of vimentin and S100A4 induced by MMP7 was inhibited with ICG-001 treatment (Figure 4G,H). The ICG-001 also restored the mRNA expression levels of the *CDH1* (E-cadherin coding gene; Figure 4G). However, it did not influence the protein level of E-cadherin in HIBEpiCs (Figure 4H). These results suggested that MMP7 promotes the nuclear translocation of β-catenin through cleavage of E-cadherin, thereby inducing EMT. Based on in vitro findings, we subsequently measured the circulating levels of sE-cadherin in BA and non-BA patients by ELISA. The results indicated a higher concentration of sE-cadherin in BA patients (Figure 4I). Furthermore, a positive correlation was observed between serum sE-cadherin and serum MMP7 concentrations (r = 0.516; *p* < 0.001; Figure 4J). Immunohistochemical analyses of β-catenin were conducted on liver tissue specimens from these patients. Compared to the non-BA group, β-catenin in the BECs of the BA group exhibited significant nuclear translocation (Figure 4K). These findings suggest that alterations in the E-cadherin/β-catenin pathway may be associated with MMP7 in BA patients.

### 2.5. MMP7 Levels Increased with Liver Fibrosis and BECs Underwent EMT in Chronic BA Mice

**Figure 5:**
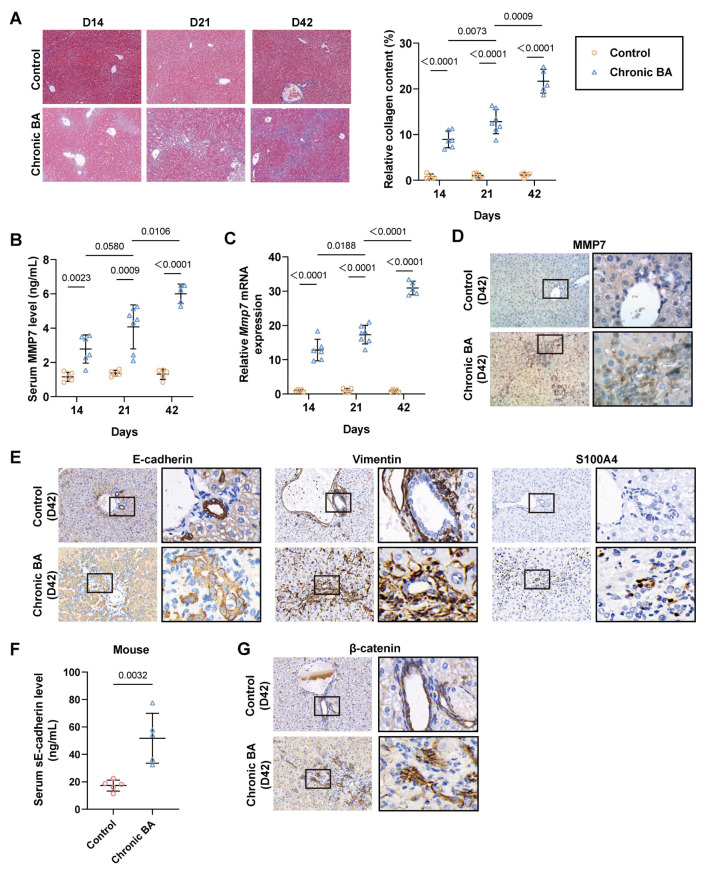
Fibrosis progression in chronic BA mice was accompanied by increased MMP7 expression and EMT. Liver and serum samples of the control group (i.p. saline) and chronic BA group (i.p. RRV+ anti-Ly6G) were collected on days 14, 21, and 42 postnatally (*n* = 5~7 mice in each group). (**A**) Representative micrograph of Masson trichrome staining of the liver from control and chronic BA mice. Quantification of collagen staining in the liver of control and chronic BA mice. Magnification: ×40. (**B**,**C**) Serum level and hepatic mRNA expression of MMP7 in control and chronic BA mice. (**D**) Representative images of immunostaining for MMP7 expression in liver (Days 42). (**E**) Representative images of immunostaining for EMT-related protein expression (including E-cadherin, Vimentin, S100A4) in liver (Days 42). (**F**) Serum level of sE-cadherin in mice (*n* = 5 mice in each group). (**G**) Representative images of immunostaining β-catenin in cholangiocytes of mice. Magnification: **left panel**: ×100; **right panel**: ×400. Data are presented as mean ± SD.

The chronic BA mouse model was first proposed in 2018 and was optimized based on the rhesus rotavirus (RRV)-induced classical BA mouse model [19,20]. In this model, the issue of severe acute inflammation and limited survival in the classical model was ameliorated through additional injections of anti-Ly6G antibodies. This modification enabled mice to develop severe liver fibrosis like BA patients. To exclude the potential interference of the anti-Ly6G antibody, we first assessed the similarity of pathological changes between chronic BA mice and BA patients through dynamic observations on days 14, 21, and 42. Masson’s trichrome staining results demonstrated significant liver fibrosis in chronic BA mice, with collagen deposition gradually increasing over time (Figure 5A). Serum levels of MMP7 exhibited a dynamic increase in chronic BA mice (Figure 5B). Additionally, the hepatic mRNA and protein levels of MMP7 were also markedly increases in the chronic BA mice compared to the control group (Figure 5C,D). The expression of EMT-related markers in the intrahepatic cholangiocytes of chronic BA mice was altered by day 42, showing decreased positive staining for E-cadherin and increased positive staining for Vimentin and S100A4 (Figure 5E). We further validated the activation of the E-cadherin/β-catenin pathway in chronic BA mice. The serum levels of sE-cadherin were also significantly elevated as compared to control mice, along with β-catenin nuclear localization (Figure 5F,G).

### 2.6. MMP7 Blockade Inhibited the Process of Liver Fibrosis and EMT in Murine BA

**Figure 6:**
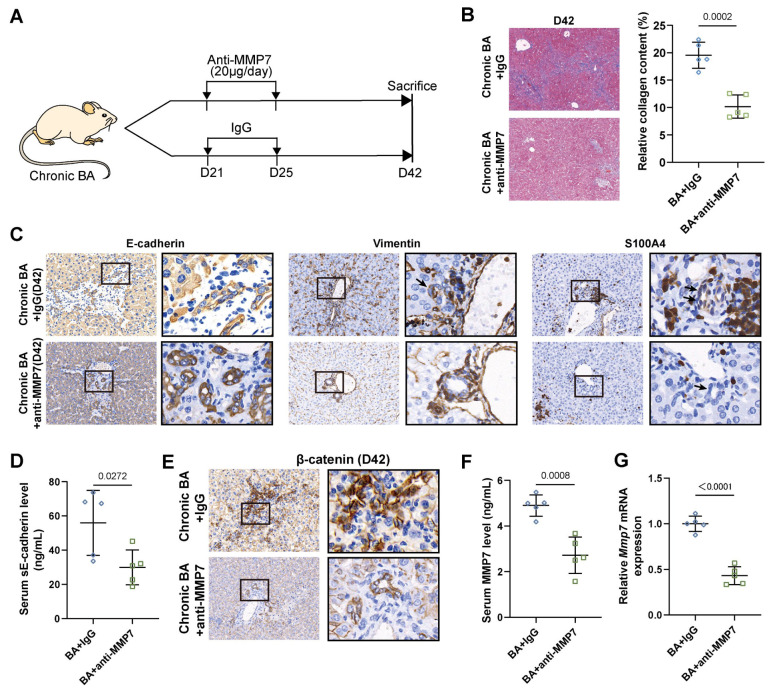
MMP7 blockade attenuated liver fibrosis and EMT procession in chronic BA mice. (**A**) Schematic representation of chronic BA mice injected with anti-MMP7 antibody or IgG on days 21–25 and liver specimens collected on day 42 (*n* = 5 mice in each group). (**B**) Representative micrographs of Masson trichrome stained liver tissue sections. Quantification of collagen staining in the liver. Magnification: ×40. (**C**) Representative images of immunostaining for EMT-related protein expression (including E-cadherin, Vimentin, S100A4) in the liver (Arrows indicate biliary epithelial cells). (**D**) Serum level of sE-cadherin in mice (*n* = 5 mice in each group). (**E**) Representative images of immunostaining β-catenin in cholangiocytes of mice. (**F**) Serum level of MMP7 in mice (*n* = 5 mice in each group). (**G**) Hepatic mRNA expression of MMP7 in mice (*n* = 5 mice in each group). Magnification: **left panel**: ×100; **right panel**: ×400. Data are presented as mean ± SD.

On the basis of the above results, we then treated chronic BA mice with a neutralizing antibody against MMP7 (Figure 6A). Chronic BA mice treated with isotype-matched IgG served as controls. MMP7 blockade significantly attenuated liver fibrosis and reduced collagen deposition (Figure 6B). Immunohistochemical analysis of EMT-related proteins showed that BECs in the treatment group exhibited higher levels of E-cadherin staining and lower levels of Vimentin and S100A4 staining compared to the control group (Figure 6C). Thus, MMP7 neutralization inhibited the EMT process in chronic BA mice. Additionally, treatment with anti-MMP7 antibodies significantly reduced serum sE-cadherin levels and decreased the nuclear translocation of β-catenin in BA mice (Figure 6D,E). Furthermore, anti-MMP7 treatment significantly reduced serum MMP7 levels in chronic BA mice (Figure 6F) and decreased liver MMP7 expression (Figure 6G).

## 3. Discussion

**Figure 7:**
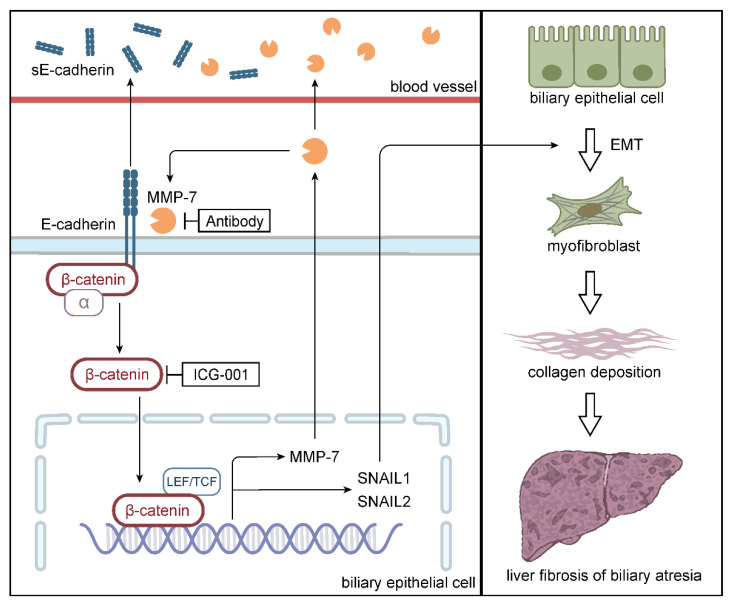
Schematic diagram of the results.

In recent years, MMP7 has garnered considerable attention due to its high value in the diagnosis of BA. However, little is known regarding its pathogenic mechanisms [21]. In this study, we demonstrate the pro-fibrotic role of MMP7 in BA. Mechanistically, MMP7 promotes EMT in intrahepatic BECs through proteolytic cleavage of E-cadherin, which subsequently induces nuclear translocation of β-catenin. Furthermore, we validated the therapeutic effect of targeting MMP7 in a chronic BA model, providing preclinical evidence for anti-fibrotic treatment (Figure 7).

During EMT, epithelial cells gradually acquire mesenchymal features while losing their epithelial characteristics. Emerging studies have shown that EMT promotes fibrosis in various organs [22]. The underlying mechanisms involve the direct conversion of epithelial cells into myofibroblasts via EMT, which subsequently participate in collagen deposition [23], as well as paracrine signaling from EMT-transformed epithelial cells that facilitates fibroblast recruitment [24,25]. In the context of BA, several studies have confirmed prominent EMT alterations in intrahepatic BECs of patients [26,27,28,29]. Here, by performing GSEA on GEO datasets and quantifying EMT markers in liver specimens from BA patients, we provide the first evidence linking MMP7 to EMT in BA. This association was further directly validated by in vitro and in vivo experiments. Collectively, our findings delineate a pathogenic MMP7-EMT-liver fibrosis axis in BA. Emerging single-cell RNA sequencing combined with spatial transcriptomics has revealed a cholangiocyte-enriched spatial niche in BA livers, which contains multiple interacting cell subtypes including activated hepatic stellate cells, macrovascular endothelial cells, TREM2-positive macrophages, and activated portal fibroblasts [30]. This finding suggests that cholangiocytes in BA may contribute to liver fibrosis through paracrine signaling, promoting the accumulation and activation of fibrosis-associated cells within their microenvironment. Due to the lack of lineage tracing studies conducted in relevant animal models, it remains unclear whether BECs directly transform into myofibroblasts in BA through EMT. Further investigations are warranted to dissect the downstream mechanisms by which EMT of BECs promotes fibrogenesis in BA.

When further investigating the specific mechanism by which MMP7 promotes EMT, we noted that the Wnt/β-catenin pathway also showed a strong correlation with MMP7 in GSEA. Nuclear translocation of β-catenin allows it to interact with transcription factors lymphoid enhancer-binding factor 1 and T cell factor, thereby activating a gene expression program that favors EMT [31,32,33]. Furthermore, studies in renal fibrosis and prostate cancer have indicated that MMP7 can disrupt the E-cadherin/β-catenin/α-catenin complex via proteolytic cleavage, promoting nuclear translocation of β-catenin [10,34]. In the present study, we demonstrated this effect of MMP7 on HIBEpiCs in vitro, and further confirmed the correlation between MMP7 and the E-cadherin/β-catenin pathway in BA patient and mouse model. Notably, we also found in vitro that MMP7 can upregulate its own expression, establishing a positive-feedback loop. This finding may explain the persistent elevation of MMP7 levels observed in some BA patients after KPE, as reported in our previous study [16]. The existence of this self-amplifying mechanism was further supported by our in vivo observations: in the chronic BA mouse model, MMP7 levels progressively increased over time, and blockade of MMP7 with a neutralizing antibody led to a significant downregulation of both circulating and hepatic MMP7, confirming the feedback regulation in the disease setting.

Notably, although ICG-001 intervention successfully restored the mRNA expression level of E-cadherin (encoded by the CDH1 gene), it failed to correspondingly restore its protein level. This seemingly paradoxical finding precisely reveals the dual regulatory mechanism of MMP7 on E-cadherin during the induction of EMT, particularly highlighting the dominant role of MMP7-mediated post-translational modification—namely, protein cleavage. MMP7 promotes the nuclear translocation of β-catenin by cleaving E-cadherin, subsequently activating EMT transcription factors including Snail1 and Snail2, which ultimately inhibit CDH1 gene expression at the transcriptional level [31]. By blocking β-catenin-mediated transcriptional repression, ICG-001 effectively restores CDH1 mRNA levels. However, the restoration of mRNA without concomitant protein recovery strongly demonstrates that in the MMP7-treated environment, a more direct and rapid mechanism of protein depletion exists beyond transcriptional regulation. As a matrix metalloproteinase, MMP7’s core function is to cleave substrate proteins. In this experimental system, MMP7 persists and directly acts on membrane-bound E-cadherin, cleaving off its extracellular domain. This physical degradation at the protein level is rapid and irreversible, directly leading to the loss of mature E-cadherin protein. In conclusion, MMP7-induced EMT is not achieved solely through classical transcriptional repression; its direct cleavage of E-cadherin (post-translational modification) serves as the initial trigger for the entire process and is crucial for maintaining the EMT phenotype.

Although this study focused on the mechanism by which MMP7 promotes EMT of BECs and subsequent liver fibrosis in BA, MMP7 may still promote liver fibrosis through other parallel mechanisms. As a matrix metalloproteinase, MMP7 degrades a broad spectrum of ECM components, such as fibronectin, gelatins, laminin, entactin, and elastin [21], and by doing so, it may contribute to ECM remodeling, creating an altered matrix microenvironment that facilitates fibrogenesis [35]. Furthermore, in hepatocellular carcinoma, MMP7 can also associate with the TGF-β signaling pathway through the MMP7/syndecan-1/TGF-β1 autocrine loop [36], suggesting that MMP7 may further promote liver fibrosis in BA by activating TGF-β1. MMP7 can also promote the activation of TNF-α and induce the expression of chemokine CXCL6, potentially contributing to maintaining the pro-fibrotic inflammatory state [37,38]. Therefore, the pro-fibrotic role of MMP7 in cholangitis may be more complex and worthy of further investigation.

The role of MMP-7 in BA has previously been explored in classic RRV-induced mouse model. The findings demonstrated that the administration of an MMP-7 antibody or inhibitor (Batimastat) prevented bile duct obstruction and alleviated liver inflammation and injury [7]. However, the high lethality of this model early in disease progression hinders the investigation of the pro-fibrotic role of MMP-7. Previous investigations into the mechanisms underlying BA-related hepatic fibrosis have predominantly utilized the bile duct ligation model [27,39]. However, such a model used adult mouse, which is not consistent with a neonatal onset of BA, and therefore can only simulate part of the characteristics. Fortunately, recent advances have been made in developing neonatal models for BA liver fibrosis, mainly including models induced by RRV reassortant and those induced by RRV combined with anti-Ly6G antibody [19,40]. Considering availability and accessibility, we chose the latter for our study. Similarly to BA patients, elevated MMP-7 levels and EMT of BECs were observed in chronic BA mice. Treatment with MMP-7 antibody significantly inhibited EMT and liver fibrosis. The findings of our study may be used as a reference for the applications of chronic BA mice model in future mechanism explorations. However, this model still has methodological limitations. The injection of anti-Ly6G antibody depletes neutrophils [19]. Given the complex role of neutrophils in inflammatory responses, tissue damage, and repair processes, their depletion may affect inflammation-related processes, including EMT and MMP7 regulation. Future studies could employ other fibrotic BA models to further validate the findings of this study while excluding the confounding effects of neutrophil depletion.

Current research indicates that BA is a highly heterogeneous disease, with significantly varied long-term outcomes following KPE [3]. Huang et al. performed cluster analysis based on the molecular profiles of liver tissues obtained at the time of KPE and classified BA patients into four molecular subtypes, which exhibit distinct pathological features and clinical outcomes [41]. This aligns with our previous findings that different patterns of MMP7 expression after Kasai surgery correspond to different prognoses [16]. Although our animal experiments preliminarily support the anti-fibrotic potential of targeting MMP7, we propose that such therapy may hold greater value for the subgroup of patients with persistently high MMP7 expression. Several highly selective MMP7 inhibitors have been developed and shown efficacy in renal fibrosis [42,43]. Further investigation is needed to identify suitable anti-MMP7 therapeutics, thereby advancing toward personalized treatment strategies for BA.

This study has several limitations. First, a formal a priori sample size calculation was not performed for either the clinical cohorts or the animal experiments. The sample size for the clinical study was determined by the availability of archived specimens, and while it is comparable to or larger than those in similar published studies, we cannot rule out the possibility of insufficient power to detect smaller effects or associations in some subgroup analyses. Additionally, while our study focuses on the mechanistic role of MMP7 in EMT-driven fibrosis, we acknowledge that several other MMP members, including MMP1, MMP2, MMP3, and MMP9, have also been reported to be dysregulated in BA, with expression patterns varying by sample type and disease stage [44,45,46]. These MMPs may act in concert or in parallel with MMP7 in the process of biliary fibrogenesis. Therefore, future studies are warranted to dissect the functional interplay among MMP family members in this context.

## 4. Materials and Methods

### 4.1. Collection and Verification of Clinical Samples

The study included patients under 1 years of age with cholestasis (serum direct bilirubin >17 µmol/L) who underwent laparoscopic/robotic surgery with intraoperative liver biopsy at our institution. All guardians provided written informed consent and agreed to comply with the study procedures. Patients were excluded if they had: (1) biliary atresia splenic malformation syndrome (BASM); (2) prior liver surgery for cholestasis (e.g., KPE) or receipt of specific anti-fibrotic/immunosuppressive therapies potentially affecting MMP7 levels; (3) contraindications to surgery/biopsy including uncorrectable coagulopathy; or (4) insufficient tissue or serum sample for analysis. According to these criteria, a total of 66 patients with BA and 39 patients with non-BA diseases (including 17 cases of cytomegalovirus hepatitis, 7 cases of choledochal cyst, 3 cases of Alagille syndrome, 3 cases of citrin deficiency, and 9 cases of cholestasis of unknown etiology) were enrolled between 2020 and 2023. Liver tissue samples were collected intraoperatively, and preoperative serum specimens were also obtained. The liver samples were sectioned in half: one was paraffin embedded after formalin fixation, while the other section was promptly frozen using liquid nitrogen and stored for later analysis. Sera were stored at −80 °C. Clinical parameters from BA and non-BA patients were collected at the time of surgery (Table 1).

The aim of this study was to determine the correlation between MMP7 expression and the degree of liver fibrosis through clinical specimen analysis, and to preliminarily explore whether EMT serves as a mediating mechanism. Accordingly, liver fibrosis stage was defined as the primary outcome, EMT score as the secondary outcome, and MMP7 expression level as the exposure factor.

The study protocol received ethical approval from the Ethical Committee of Union Hospital, Tongji Medical College, Huazhong University of Science and Technology (Ethics Approval Number: 2020S029) on 29 September 2020. The study was registered in the Chinese Clinical Trial Registry [ChiCTR1900028456, registered 22 December 2019, http://www.chictr.org.cn/ (accessed on 5 January 2020)].

### 4.2. Single-Gene Correlation-Based Gene Set Enrichment Analysis

The GSE15235 and GSE46960 datasets were retrieved from the Gene Expression Omnibus (GEO) database [https://www.ncbi.nlm.nih.gov/geo/ (accessed on 11 March 2023)]. From GSE46960, data from 64 BA patients were extracted. For GSE15235, data from 41 BA patients were included after excluding 6 cases with BASM. Pearson correlation coefficients between MMP7 and all other genes were calculated across all samples. Genes were then ranked based on their correlation coefficients. The distribution of correlation coefficients for all genes, along with the top positively and negatively correlated genes, was generated using the R package (R version 2.15.0) “ggplot2” to illustrate the overall correlation pattern. Using the ranked list, pre-defined HALLMARK gene sets from the MSigDB database were tested for enrichment with the GSEA algorithm implemented in the R package “clusterProfiler”. Significantly enriched pathways (adjusted *p* value < 0.05) were identified and visualized using a bar plot ordered by Normalized Enrichment Score.

### 4.3. Cell Culture and Treatment

Human intrahepatic biliary epithelial cells (HIBEpiCs), obtained from ScienCell Research Laboratories (California, USA), were cultured according to the provided instructions. The activation of recombinant human MMP7 protein (R&D Systems, Minneapolis, MN, USA) was achieved through incubation with 4-aminophenylmercuric acetate (APMA; Sigma, St. Louis, MO, USA). HIBEpiCs were exposed to varying concentrations (0, 20, 40, and 80 ng/mL) of the activated MMP7 protein for a duration of 24 h. For inhibitory experiments, 25 μM ICG-001 (ApexBio, Houston, TX, USA) was added along with the MMP7 protein to cells, and they were co-cultured for 24 h. DMSO was added to control cultures as a vehicle control.

### 4.4. The Experimental Model of Chronic BA

The BALB/c mice (8–12 weeks old) were obtained from Biont (Wuhan, China). The mice were kept in a controlled environment that met specific pathogen-free standards, with a constant temperature of 25 °C, access to ample water, free intake, and a 12/12 h circadian rhythm. Chronic BA was induced according to protocols published previously [19,20]. Briefly, newborn mice were injected intraperitoneally with 20 μL of 1.5 × 106 PFU/mL rhesus rotavirus (RRV; kindly provided as a gift by Prof. CL Mack, University of Colorado, Denver, USA) or 0.9% saline as a vehicle control within the first 24 h of birth. To prolong the lifespan of mice and thus obtain a fibrotic phenotype, additional intraperitoneal injections of anti-Ly6G antibody were performed a few times (including 4 h before RRV injection and days 4, 8, and 12 after RRV injection). Mice were then sacrificed in batches on days 14, 21, and 42, and livers and sera were collected. For MMP7 neutralization experiments, chronic BA mice received daily intraperitoneal injections with 20 µg of goat-derived anti-MMP7 antibody or normal goat IgG (R&D System) as control during days 21 to 25. Mouse livers and sera were harvested on day 42 for analysis. The Animal Protection and Use Committee at Tongji Medical College, Huazhong University of Science and Technology granted ethical approval for all experimental procedures (IACUC Number: 2882). To minimize suffering, animals were anesthetized with pentobarbital sodium (50 mg/kg, i.p.) before blood samples collection and then euthanized with an overdose of pentobarbital sodium (200 mg/kg, i.p.).

### 4.5. Enzyme-Linked Immunosorbent Assay (ELISA)

The serum concentration of MMP7 was detected by the Human Total MMP7 Quantikine ELISA Kit (R&D System) and the Mouse MMP7 ELISA Kit (Cloud-Clone Corp, Wuhan, China). The concentrations of soluble E-cadherin (sE-cadherin) in cell culture supernatants and serum were measured using the Human E-Cadherin Quantikine ELISA Kit (R&D System) and the Mouse E-Cadherin ELISA Kit (Abcam, Cambridge, UK). ELISAs were performed following the manufacturer’s instructions.

### 4.6. Histology and Immunohistochemical Analysis

Immunohistochemical staining was conducted following established protocols as described previously [47]. The primary antibodies used were as follows: Rabbit Anti-Human MMP7 (Abcam), Rabbit Anti-Mouse MMP7 (GeneTex, Irvine, CA, USA), Rabbit Anti-Human E Cadherin, Rabbit Anti-Mouse E Cadherin, Rabbit Anti-Human/Mouse Vimentin, Rabbit Anti-Human/Mouse S100A4 (all from Abcam, Cambridge, UK), Rabbit Anti-Human/Mouse β-catenin (Cell Signaling Technology, Danvers, MA, USA). For the detection of liver fibrosis, Masson trichrome staining of liver tissue was performed using Masson’s Trichrome Staining Kit (Servicebio, Wuhan, China). The METAVIR scoring system was used for liver fibrosis and inflammation scoring in human BA [48]. The sections were reviewed and scored by two independent observers, and a consensus was reached. For analyses involving BECs, at least five portal areas were examined in each slice. Following microscopic photography, the expression intensity of MMP7 (calculated as IOD/area) and the area of collagen fiber deposition were quantified using the ImageJ software (version 1.53c).

### 4.7. EMT Score

The staining of EMT-related proteins was graded according to the intensity of staining and the proportion of stained BECs [17]. E-cadherin staining was graded as preserved, reduced, or lost, corresponding to scores of 0, 1, and 2, respectively. Preserved refers to ≥90% of BECs showing strongly membranous staining, reduced refers to <90% but ≥50% of BECs show positive membranous staining, and lost refers to <50% of BECs showing positive membranous staining. For vimentin and S100A4, immunohistochemical staining was graded as absent, mild, or severe, corresponding to scores of 0, 1, and 2, respectively. Absent refers to no positive staining of BECs, mild refers to ≤20% of BECs were weakly positive staining, and severe refers to >20% strongly positive staining. The EMT score is calculated as the cumulative protein levels of E-cadherin, vimentin, and S100A4 in the BECs, ranging from 0 to 6.

### 4.8. RT-PCR

The liver tissue and cells were subjected to total RNA extraction, followed by cDNA synthesis using the EntiLink™ 1st Strand cDNA Synthesis Kit (ELK Biotechnology, Wuhan, China) as per the manufacturer’s protocols. RT-PCR was conducted on a StepOne™ Real-Time PCR machine (Thermo Fisher Scientific, Waltham, MA, USA) with the EnTurbo™ SYBR Green PCR SuperMix kit (ELK Biotechnology). The provided guidelines are referenced for the preparation of the reaction system. Supplementary Tables S3 and S4 contain all gene primer pairs used.

### 4.9. Western Blotting

The HIBEpiCs were lysed using a cell lysis buffer, and the resulting total protein was quantified utilizing a BCA protein assay kit (Beyotime, Shanghai, China). The NE-PER Nuclear and Cytoplasmic Extraction Reagents were employed to obtain cytoplasmic and nuclear protein fractions, following the manufacturer’s protocols (Thermo Fisher Scientific). Equal amounts of cellular protein were separated using 10% or 12% SDS-PAGE, transferred onto PVDF membranes, and blocked with 5% skimmed milk. The primary antibodies used for the incubation of the membranes included those targeting E-cadherin, Vimentin, S100A4 (Abcam), β-catenin (Cell Signaling Technology), Histone H3, and GAPDH (Proteintech, Wuhan, China). The incubation was carried out overnight at a temperature of 4 °C. After washing and incubation with a secondary antibody (MCE, Monmouth Junction, NJ, USA), the bands were identified by ECL reagents (Abclonal, Wuhan, China), and visualized using the BG-gdsAUTO710MINI imaging system (Baygene, Beijing, China).

### 4.10. Immunofluorescence

The HIBEpiCs were fixed using a 4% paraformaldehyde solution for a duration of 15 min. Subsequently, permeabilization was carried out at room temperature utilizing a 0.5% Triton X-100 solution (excluding membrane proteins). After washing, the cells underwent blocking with a 5% BSA solution at room temperature for one hour. Thereafter, incubation was performed overnight at 4 °C employing a primary antibody targeting β-catenin (Cell Signaling Technology). The cells were then thoroughly washed and incubated with a CY3-conjugated anti-rabbit secondary antibody (MCE). The nuclei were counterstained with DAPI. Finally, an anti-fluorescence quenching agent was utilized to seal the stained cells before visualization under a fluorescence microscope (Olympus IX73, Tokyo, Japan).

### 4.11. TOP/FOP Flash Assay

The TOP/FOP Flash test was conducted following the guidelines provided by the manufacturer. (Upstate Biotechnology, Lake Placid, NY, USA). HIBEpiCs were plated in 24-well plates and then transfected with Top-flash or Fop-flash plasmid (Upstate) plus pRL-CMV plasmid (Promega, Madison, WI, USA). The β-catenin transcriptional activity was assessed by measuring luciferase activity using a Dual Luciferase Kit (Promega) after two days. The transfection efficiency was normalized to pRL-CMV activity.

### 4.12. Statistical Analysis

The correlation was assessed using Spearman’s correlation analysis. Survival was analyzed with Kaplan–Meier survival plots and differences were tested by Log-rank test. The analysis of discrete variables was performed by the chi-square test or Fisher’s exact test. Normality of data distribution was assessed using the Shapiro–Wilk test. For comparisons between two groups, Student’s *t*-test was applied for normally distributed data, while the Mann–Whitney U test was used for non-normally distributed data. All experiments were performed independently for a minimum of three repetitions. Statistical analysis was performed using SPSS 26.0 software, and a significance level of *p* < 0.05 was adopted.

## 5. Conclusions

In summary, our research demonstrates that MMP7 promotes BA liver fibrosis by inducing EMT in BECs. Furthermore, both in vitro and in vivo assays confirm that MMP7 facilitates EMT via the E-cadherin/β-catenin pathway. Hence, these findings establish a proof of concept for MMP7 as a pathogenic mediator and a potential therapeutic target for liver fibrosis in BA.

## Figures and Tables

**Table 1:** Patient demographics and baseline clinical characteristics.

	BA (*n* = 66)	Non-BA (*n* = 39)	*p* Value
Age at operation, median (IQR), days	56 (43–74)	69 (43–110)	0.14
Sex, *n* (%)			0.757
Female	38 (58.5%)	24 (61.5%)	
Male	27 (41.5%)	15 (38.5%)	
ALT, median (IQR), U/L	125 (64–204)	89 (43–143)	0.092
AST, median (IQR), U/L	153 (105–239)	136 (70–242)	0.165
GGT, median (IQR), U/L	348 (220–686)	251 (132–378)	0.003
DBIL, median (IQR), μmol/L	93.9 (71.3–117.0)	73.3 (53.4–107.5)	0.009
TBIL, median (IQR), μmol/L	145.4 (119.0–179.9)	113.4 (89.9–165.9)	0.012
ALP, median (IQR), U/L	584.3 (328.6–774.8)	488.6 (332.1–718.4)	0.185
TBA, median (IQR), μmol/L	100.1 (72.6–133.2)	93.5 (61.5–112.7)	0.114
Fibrosis stage, *n* (%)			<0.001
F0	0	14	
F1	10	13	
F2	20	11	
F3	23	1	
F4	13	0	
Inflammation stage, *n* (%)			<0.001
A0	0	4	
A1	8	22	
A2	36	13	
A3	22	0	

Abbreviations: BA, biliary atresia; IQR, interquartile range; ALT, alanine aminotransferase; AST, aspartate aminotransferase; GGT, gamma-glutamyl transpeptidase; DBIL, direct bilirubin; TBIL, total bilirubin; ALP, alkaline phosphatase; TBA, total bile acids.

## Data Availability

The datasets used and/or analyzed during the current study are available from the corresponding author on reasonable request.

## Supplementary Materials

The following supporting information can be downloaded at: https://www.mdpi.com/article/10.3390/ijms27052209/s1.

## Abbreviations

BA	Biliary atresia
MMP-7	Matrix metalloproteinase-7
EMT	Epithelial-to-mesenchymal transition
BECs	Biliary epithelial cells
KPE	Kasai portoenterostomy
ECM	Extracellular matrix
GEO	Gene expression omnibus
GSEA	Gene set enrichment analysis
HIBEpiCs	Human bile duct epithelial cells
COL1A1	Collagen type I alpha 1
ACTA2	Alpha actin 2
TGFB1	Transforming growth factor beta 1
TIMP1	Tissue inhibitor of metalloproteinases-1
TNF-α	Tumor necrosis factor-α
BDL	Bile duct ligation
