# Supplementary material for: Matrix Metalloproteinase 7 Mediates Epithelial–Mesenchymal Transition to Promote Liver Fibrosis Through E-cadherin/β-catenin Pathway in Biliary Atresia
Source: Int J Mol Sci. 2026 Feb 26;27(5):2209. doi: 10.3390/ijms27052209 (PMC12986156; doi:10.3390/ijms27052209)
Supplement: Supplementary file 1 [file ijms-27-02209-s001.zip › ijms-4113047-supplementary.pdf]

Matrix metalloproteinase 7 mediates epithelial-mesenchymal transition to promote liver fibrosis through E-cadherin/β-catenin pathway in biliary atresia

Authors and Affiliations: Liying Rong[1#], Jingfeng Tang[2#], Xiangyang Li [1], Mengxin Zhang[1], Shuiqing Chi[1], Yun Zhou[1], Xi Zhang[1], Guoqing Cao[1], Yibo Li[1*], Shao-tao Tang[1*]

[1] Department of Pediatric Surgery, Union Hospital, Tongji Medical College, Huazhong University of Science and Technology, Wuhan, China

[2] Department of Traumatology and Emergency Surgery, Union Hospital, Tongji Medical College, Huazhong University of Science and Technology, Wuhan, China

* Corresponding author:

Shao-tao Tang

Department of Pediatric Surgery, Union Hospital, Tongji Medical College, Huazhong University of Science and Technology, Wuhan, China

Tel: (+86) 13720313268, (+86) 27 85350752

Email: tshaotao83@126.com; tshaotao83@hust.edu.cn

Yibo Li

Department of Pediatric Surgery, Union Hospital, Tongji Medical College, Huazhong University of Science and Technology, Wuhan, China

Tel: (+86) 15090181078

Email： liyibo19@sina.cn

[#] These authors contributed equally: Liying Rong and Jingfeng Tang

Supplementary Figure S1. Hepatic mRNA expression of fibrotic marker genes increased in BA patients. Relative mRNA levels of the hepatic fibrotic marker genes, including *COLIA1*, *ACTA2*, *TGFB1*, and *TIMP1* in livers of BA patients (n = 66) and non-BA controls (n = 39).

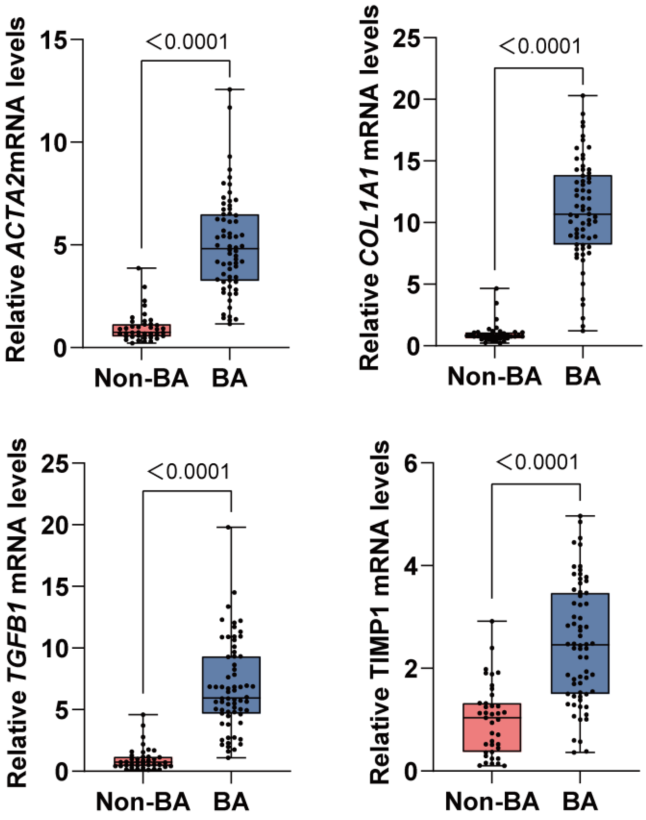

Supplementary Figure S2. MMP7 expression in BA patients stratified by hepatic inflammatory grade. MMP7 levels were compared among BA patients grouped according to METAVIR inflammation grades (A1, A2, and A3), including serum MMP7 concentrations measured by ELISA, hepatic MMP7 mRNA expression assessed by qRT-PCR, and MMP7 protein expression in liver tissues quantified by immunohistochemistry (IOD/area).

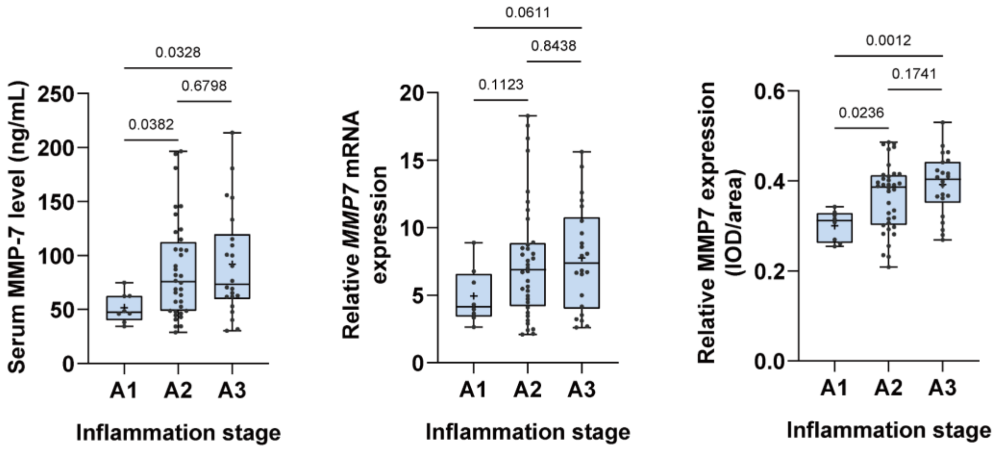

Supplementary Table S1. GSEA results for MMP7-correlated genes in BA (GSE15235).

Pathway Name	NES	p value	p.adjust	q value
HALLMARK_EPITHELIAL_MESENCHYMAL_TRANSITION	3.388636	1E-10	8.33E-10	3.51E-10
HALLMARK_HEME_METABOLISM	-2.53706	1E-10	8.33E-10	3.51E-10
HALLMARK_UV_RESPONSE_DN	2.524238	1E-10	8.33E-10	3.51E-10
HALLMARK_E2F_TARGETS	-2.40667	1E-10	8.33E-10	3.51E-10
HALLMARK_OXIDATIVE_PHOSPHORYLATION	-2.32075	1E-10	8.33E-10	3.51E-10
HALLMARK_G2M_CHECKPOINT	-2.20303	1E-10	8.33E-10	3.51E-10
HALLMARK_XENOBIOTIC_METABOLISM	-2.09186	3.71E-09	2.65E-08	1.12E-08
HALLMARK_FATTY_ACID_METABOLISM	-1.97671	5.02E-07	3.14E-06	1.32E-06
HALLMARK_WNT_BETA_CATENIN_SIGNALING	2.357173	1.1E-06	6.09E-06	2.56E-06
HALLMARK_ANGIOGENESIS	2.325417	1.55E-06	7.13E-06	3.00E-06
HALLMARK_TGF_BETA_SIGNALING	2.239921	1.57E-06	7.13E-06	3.00E-06
HALLMARK_BILE_ACID_METABOLISM	-2.00495	8.84E-06	3.68E-05	1.55E-05
HALLMARK_APICAL_JUNCTION	1.805927	1.05E-05	4.03E-05	1.70E-05
HALLMARK_KRAS_SIGNALING_UP	1.746805	3.05E-05	0.00010893	4.59E-05
HALLMARK_HEDGEHOG_SIGNALING	2.13855	3.29E-05	0.00010968	4.62E-05
HALLMARK_MTORC1_SIGNALING	-1.71984	4.25E-05	0.00013268	5.59E-05
HALLMARK_ESTROGEN_RESPONSE_EARLY	1.724462	4.89E-05	0.0001437	6.05E-05
HALLMARK_MYC_TARGETS_V2	-1.98852	9.26E-05	0.00025716	0.000108279
HALLMARK_SPERMATOGENESIS	-1.77886	0.000126	0.00033228	0.000139906
HALLMARK_CHOLESTEROL_HOMEOSTASIS	-1.88997	0.000269	0.00067363	0.000283636
HALLMARK_KRAS_SIGNALING_DN	-1.60868	0.00037	0.00088192	0.000371333
HALLMARK_PEROXISOME	-1.69221	0.000874	0.0019872	0.000836714
HALLMARK_ADIPOGENESIS	-1.50946	0.001669	0.00362885	0.001527935
HALLMARK_NOTCH_SIGNALING	1.676977	0.006005	0.01250971	0.005267246

HALLMARK_DNA_REPAIR	-1.47977	0.008497	0.01699399	0.007155366
HALLMARK_REACTIVE_OXYGEN_SPECIES_PATHWAY	-1.59429	0.014728	0.02832227	0.011925167
HALLMARK_MYOGENESIS	1.343227	0.017275	0.03199022	0.013469564
HALLMARK_HYPOXIA	1.327907	0.019493	0.03480969	0.014656713
HALLMARK_P53_PATHWAY	1.297268	0.0271	0.04516746	0.019017878
HALLMARK_MITOTIC_SPINDLE	1.287478	0.026807	0.04516746	0.019017878

Supplementary Table S2. GSEA results for MMP7-correlated genes in BA (GSE46960).

Pathway Name	NES	p value	p.adjust	q value
HALLMARK_EPITHELIAL_MESENCHYMAL_TRANSITION	3.354688991	1.00E-10	7.14E-10	2.11E-10
HALLMARK_UV_RESPONSE_DN	3.138720982	1.00E-10	7.14E-10	2.11E-10
HALLMARK_E2F_TARGETS	-2.64287765	1.00E-10	7.14E-10	2.11E-10
HALLMARK_OXIDATIVE_PHOSPHORYLATION	-2.51837904	1.00E-10	7.14E-10	2.11E-10
HALLMARK_FATTY_ACID_METABOLISM	-2.46657025	1.00E-10	7.14E-10	2.11E-10
HALLMARK_XENOBIOTIC_METABOLISM	-2.35130472	1.00E-10	7.14E-10	2.11E-10
HALLMARK_G2M_CHECKPOINT	-2.2530675	1.00E-10	7.14E-10	2.11E-10
HALLMARK_APICAL_JUNCTION	2.137644695	2.61E-09	1.63E-08	4.80E-09
HALLMARK_BILE_ACID_METABOLISM	-2.27028386	5.32E-09	2.96E-08	8.72E-09
HALLMARK_TGF_BETA_SIGNALING	2.490998665	1.48E-08	7.39E-08	2.18E-08
HALLMARK_CHOLESTEROL_HOMEOSTASIS	-2.20894268	3.26E-07	1.48E-06	4.36E-07
HALLMARK_ANGIOGENESIS	2.383320891	6.98E-07	2.86E-06	8.44E-07
HALLMARK_MTORC1_SIGNALING	-1.93470218	7.45E-07	2.86E-06	8.44E-07
HALLMARK_TNFA_SIGNALING_VIA_NFKB	1.915628603	8.16E-07	2.91E-06	8.59E-07
HALLMARK_KRAS_SIGNALING_UP	1.896634769	2.00E-06	6.66E-06	1.96E-06
HALLMARK_MITOTIC_SPINDLE	1.932585472	2.42E-06	7.57E-06	2.23E-06
HALLMARK_HEDGEHOG_SIGNALING	2.325009602	2.58E-06	7.60E-06	2.24E-06

HALLMARK_ADIPOGENESIS	-1.79802284	1.01E-05	2.80E-05	8.25E-06
HALLMARK_PEROXISOME	-1.98009272	1.32E-05	3.47E-05	1.02E-05
HALLMARK_HEME_METABOLISM	-1.77740695	3.41E-05	8.52E-05	2.51E-05
HALLMARK_DNA_REPAIR	-1.80961333	5.24E-05	0.000119922	3.53E-05
HALLMARK_ESTROGEN_RESPONSE_EARLY	1.713167062	5.28E-05	0.000119922	3.53E-05
HALLMARK_MYOGENESIS	1.731796288	6.96E-05	0.000151289	4.46E-05
HALLMARK_IL2_STAT5_SIGNALING	1.670991753	0.000126252	0.000263025	7.75E-05
HALLMARK_INTERFERON_ALPHA_RESPONSE	-1.83149961	0.000150146	0.000300292	8.85E-05
HALLMARK_MYC_TARGETS_V2	-1.9659806	0.000222925	0.000428703	0.000126354
HALLMARK_APOPTOSIS	1.734627536	0.00037756	0.000699186	0.000206076
HALLMARK_NOTCH_SIGNALING	1.839312155	0.001512724	0.002701292	0.00079617
HALLMARK_PROTEIN_SECRETION	1.626116561	0.003839452	0.006619746	0.001951083
HALLMARK_WNT_BETA_CATENIN_SIGNALING	1.715234065	0.00908648	0.015144134	0.004463534
HALLMARK_HYPOXIA	1.407412193	0.009787232	0.015785858	0.004652674
HALLMARK_GLYCOLYSIS	-1.36835916	0.014837473	0.023183552	0.006833047
HALLMARK_REACTIVE_OXYGEN_SPECIES_PATHWAY	-1.53130629	0.018776111	0.028201453	0.008312007
HALLMARK_COAGULATION	1.410643218	0.019176988	0.028201453	0.008312007
HALLMARK_ANDROGEN_RESPONSE	1.438976701	0.019792981	0.028275688	0.008333887
HALLMARK_KRAS_SIGNALING_DN	-1.29816925	0.026510578	0.036820247	0.010852283

Supplementary Table S3. Nucleotide sequences of the primers used for RT-PCR (human gene)

Human gene	Primer Sequence 5' to 3'
MMP7	
Forward primer	GGGGACTCCTACCCATTTGAT
Reverse Primer	CGTCCAGCGTTCATCCTCAT
COL1A1	
Forward primer	AAAGATGGACTCAACGGTCTC
Reverse Primer	CATCGTGAGCCTTCTCTTGAG
ACTA2	
Forward primer	CCGGGACTAAGACGGGAATC
Reverse Primer	TTGTCACACACCAAGGCAGT
TGFB1	
Forward primer	GTTCAGGTACCGCTTCTCGG
Reverse Primer	CCTGATCGCCTCCTTCATTT
TIMP1	
Forward primer	CACTGTTGGCTGTGAGGAA
Reverse Primer	AAGGTGACGGGACTGGAA
SNAI1	
Forward primer	CCAGACCCACTCAGATGTCA
Reverse Primer	GGACTCTTGGTGCTTGTGGA
SNAI2	
Forward primer	GCCAAACTACAGCGAACTGG
Reverse Primer	GAGAGAGGCCATTGGGTAGC
CDH1	
Forward primer	GAGAAACAGGATGGCTGAAGG
Reverse Primer	TGAGGATGGTGTAAGCGATGG
VIM	
Forward primer	AGTCCACTGAGTACCGGAGAC
Reverse Primer	CATTTCACGCATCTGGCGTTC
S100A4	
Forward primer	TCTTTCTTGGTTTGATCCTG
Reverse Primer	GCATCAAGCACGTGTCTGAA
ACTB	
Forward primer	CGGGAAATCGTGCGTGAC
Reverse Primer	CATTGCCAATGGTGATGACCT

Supplementary Table S4. Nucleotide sequences of the primers used for RT-PCR (mouse gene)

Mouse gene	Primer Sequence 5' to 3'
Mmp7	
Forward primer	CTCGCTCCTGGAAGATGGTGAT
Reverse Primer	TGGCACCACCGAGGACTATGAC
Actb	
Forward primer	AAGGCCAACCGCGAGAA
Reverse Primer	CCTCGTAGATGGGCACA
